# Epidemiology and Genetic Diversity of Hepatitis B Virus and Hepatitis Delta Virus Infection in Indigenous Communities in Colombia

**DOI:** 10.3390/microorganisms11071739

**Published:** 2023-07-03

**Authors:** Melissa Montoya-Guzman, Jaime Martinez, Diana Castro-Arroyave, Carlos Rojas, Maria-Cristina Navas

**Affiliations:** 1 Grupo Gastrohepatología, Facultad de Medicina, Universidad de Antioquia, Calle 70 No. 52-21, Medellín 050010, Colombia; melissa.montoya@udea.edu.co; 2 Grupo Epidemiología, Facultad Nacional de Salud Pública, Universidad de Antioquia, Calle 70 No. 52-21, Medellín 050010, Colombia; 3 Grupo de Estudio en Pedagogía, Infancia y Desarrollo Humano (GEPIDH), Facultad de Educación, Universidad de Antioquia, Calle 70 No. 52-21, Medellín 050010, Colombia

**Keywords:** HBV, HDV, infection, viral genotypes, indigenous communities, Colombia

## Abstract

Despite the universal vaccination program, there are still regions and territories with a high prevalence of Hepatitis B Virus infection (HBV), such as the Amazon basin, where several indigenous communities live. Additionally, Hepatitis Delta Virus (HDV) is a defective that requires the hepatitis B surface antigen (HBsAg) for the assembly and release of de novo viral particles. Therefore, hepatitis D could be the result of HBV/HDV coinfection or HDV superinfection in individuals with chronic hepatitis B. Among the high prevalence HDV populations are indigenous communities of America. This study aims to describe and characterize the frequency of HBV and HDV infection, viral genotypes and HBV immune escape mutants in indigenous populations from different regions of Colombia. The diagnosis of hepatitis B and hepatitis D was confirmed by serological markers. Moreover, the HBV and HDV genome were amplified by PCR and RT-PCR, respectively, and, subsequently, the phylogenetic analysis was performed. We characterized 47 cases of chronic hepatitis B, 1 case of reactivation and 2 cases of occult hepatitis B infection (OBI). Furthermore, a high prevalence of HDV infection was identified in the study population (29.33%, 22/75) and the circulation of several HBV genotypes and subgenotypes (F1b, F3, F4, and D). Interestingly, this is the first report of the HDV genotype I circulation in this country. These findings demonstrated that HBV and HDV infections are still public health problems in indigenous communities in Colombia.

## 1. Introduction

The Hepatitis B Virus (HBV) is an etiologic agent of acute and chronic infection with the risk of progression to liver cirrhosis and hepatocellular carcinoma (HCC) [1]. The World Health Organization (WHO) estimates a global prevalence of 296 million cases of chronic HBV infection and 820,000 deaths related to HBV per year [2].

Furthermore, Occult HBV Infection (OBI) has been also described [3]. This infection is characterized by a low viral load (<200 IU/mL) and undetectable hepatitis B surface antigen (HBsAg); in addition, antibodies anti-hepatitis B Core protein (Anti-HBc) are detected in most OBI cases [3].

The HBV belongs to the family *Hepadnaviridae*, genus *Orthohepadnavirus* [4]. Ten HBV genotypes (A–J), with a genetic diversity of more than 8%, and 40 subgenotypes with a genetic divergence of more than 4% have been described [5]. HBV genotypes F and A are the most prevalent reported in different populations in Colombia; although genotypes C, D, E and genotype G have been also described [6,7,8,9].

According to the Colombian epidemiological surveillance system, the average incidence of hepatitis B was between 2.5–4.68 per 100,000 inhabitants in the last decade (2011–2021) [10]. However, some states such as Amazonas and Guaviare have been characterized by a high incidence of hepatitis B in the last decades [10].

On the other hand, Hepatitis Delta Virus (HDV) is an RNA virus that depends on HBV as helper virus. Indeed, HDV requires the HBsAg for the assembly and release of de novo viral particles. HDV is classified in the family *Kolmioviridae*, genus *Deltavirus*, [11,12].

There are two types of infections of HBV/HDV: (i) coinfection is the simultaneous HBV and HDV acute infection, which is mainly associated with viral clearance; (ii) HDV superinfection in chronically HBV-infected individuals that evolves in most cases to chronic hepatitis Delta with liver failure in 7–15% of patients [11,13].

The global prevalence of HDV infection has been estimated among 12 million cases (4.5 %) [14] and 48–60 million cases (13.02%) in HBsAg seropositive individuals [15]. Furthermore, there are some endemic regions over the world including the Amazon Basin [16].

Eight HDV genotypes have been characterized with a genetic divergence of 40% [11,17]. The most prevalent HDV genotype in South America is III [18]. However, HDV Genotype I has also been identified in the region, and the circulation of HDV genotype VIII has recently been reported in Brazil [19,20].

Some studies carried out in Colombia demonstrated a high prevalence of hepatitis B and hepatitis Delta in indigenous communities from Amazonas state [21,22,23,24]. However, the current situation in indigenous communities from other regions of the country is unknown. This study aims to describe and characterize the frequency of HBV and HDV infection, the viral genotypes and the HBV immune escape mutants in samples obtained from indigenous individuals of different regions of Colombia.

## 2. Materials and Methods

### 2.1. Study Population

The study area is located in Amazonas, Guaviare (south of the country, Amazonia region), Antioquia (Northwest) and La Guajira (extreme northeast) states in Colombia. Cases of hepatitis B notified to the Colombian epidemiological surveillance system in indigenous people > 18 years old during the period 2015–2022 were included in the study. Informed consent was obtained according to requirements of the Ethics Committee. The indigenous who speak Spanish and agreed to participate were informed of the risks and benefits. A standardized form was used to interview the individuals.

Blood samples were collected in vacuum tubes by venipuncture; then, centrifuged and the serum samples stored at local laboratories were subsequently sent to the Gastrohepatologia Group of the University of Antioquia in a primary container and a secondary container leak-proof with dry ice. The samples were stored at −70 °C.

This study follows the international guidelines for research with human beings expressed in the Declaration of Helsinki of the World Medical Association in its latest version. The Ethics Committee of the National School of Public Health of the University of Antioquia approved the project. The results of the study were shared with the health authorities of each state.

### 2.2. Serological Markers of HBV and HDV Infection

HBsAg, anti-HBc IgM and Total markers detection were performed in serum samples by chemiluminescence. The ALT and AST serum levels were quantified by UV Test according to the modified IFCC (International Federation of Clinical Chemistry and Laboratory Medicine). The samples HBsAg+/Anti-HBc+ and serum levels ALT > 49 IU/mL and/or AST > 34 IU/mL were analyzed for the hepatitis B e antigen (HBeAg) and antibodies of the anti-e antigen (anti-HBe) markers by chemiluminescence. In addition, the total anti-HDV antibodies were determined using a competitive ELISA (DIA.PRO) in all serum samples by duplicate.

### 2.3. HBV Genome Detection

The total DNA was obtained from 200 µL of the serum sample obtained from cases using a commercial kit (QIAamp DNA Blood mini kit, QIAGEN, Hilden, Germany). Amplification conditions, primers and cycling were modified from protocols previously published (Supplementary Table S1) [21,22,25]. The first round included 1× buffer, 4 mM MgCl_2_, 0.2 mM dNTPs (Promega, Madison, WI, USA), 0.4 µM primers YS1 and YS2, and 1.25 U of Taq DNA polymerase (Thermo Scientific, Waltham, MA, USA). The cycling was 94 °C for 3 min, followed by 35 cycles of 94 °C for 45 s, 53 °C for 1 min, extension at 72 °C for 1.30 min, and 72 °C for 6 min. For the second round, 1× buffer, 2 mM MgCl_2_, 0.2 mM dNTPs (Promega), 0.4 µM primers S3S and S3as and 1.25 U Taq DNA polymerase (Thermo Scientific, Waltham, MA, USA) were used. There was cycling with 94 °C for 4 min, and this was followed by 35 cycles of 94 °C for 1 min, 50 °C for 1:15 min and an extension at 72 °C for 1:30 min, with a final extension at 72 °C for 5 min.

Amplification conditions and cycling for HBV PreS1 and PreS2 regions were modified from protocols previously published using primers P1–P2 in the first round [21,26] (Supplementary Table S1). The 1× buffer, 1.5 mM MgCl_2_, 0.2 mM dNTPs (Promega), 1 µM primers P1 and P2 and 5U Taq DNA polymerase (Thermo Scientific) were used. Touchdown cycling was designed at 94 °C for 4 min, with 10 cycles at 94 °C for 40 s, 55 °C for one min, 72 °C for 3 min, 10 cycles of 94 °C for 40 s, 60 °C for 1 min, 72 °C for 5 min, 10 cycles of 94 °C for 40 s, 62 °C for 1 min, 72 °C for 7 min, 10 cycles of 94 °C for 40 s, 64 °C for 1 min, 72 °C for 9 min and a final extension of 72 °C for 10 min.

The second round was performed as previously reported [21,26]. For the PreS1 region, 1× buffer, 1.5 mM MgCl_2_, 0.2 mM dNTPs (Promega), 0.4 µM 2440p-58n primers [21,26] (Supplementary Table S1) and 1.5 U Taq DNA polymerase (Thermo Scientific) were used. Cycling was at 95 °C for 3 min, with 35 cycles of 95 °C for 40 s, 55 °C for 30 s and extension at 72 °C for 1 min, with a final extension at 72 °C for 10 min. For the PreS2 region, 1× buffer, 1.5 mM MgCl_2_, 0.2 mM dNTPs (Promega), 0.4 µM p3006f-p213r primers [21] and 1.5 U Taq DNA polymerase (Thermo Scientific) at 95 °C for 3 min was followed by 35 cycles of 95 °C for 40 s, 59 °C for 30 s, and an extension at 72 °C for 1 min, with a final extension of 72 °C for 10 min.

### 2.4. HDV Genome Detection

Viral RNA was obtained from serum samples using a commercial kit (QIAamp Viral RNA Mini Kit, QIAGEN). Then, the cDNA was synthesized using the primer 1302D [22] 0.67 µM and the enzyme RevertAid RT (Thermo Scientific) following the suggested protocol. Amplification conditions and cycling were modified from protocols previously published [22]. The first round was carried out with 1× buffer, 2 mM MgCl_2_, 0.2 mM dNTPs (Promega), 0.5 µM using the primers 8531U, and 1302D [22] (Supplementary Table S1) and Taq DNA polymerase (Thermo Scientific) 1.25. There were cycles of 95 °C for 2 min, followed by 35 cycles of 95 °C for 30 s, 56 °C for 30 s, extension at 72 °C for 1 min, and 72 °C for 10 min. The second round reaction was carried out with 1× buffer, 2 mM MgCl_2_, 0.2 mM dNTPs (Promega), using the primers HDV-E and HDV-A at 0.5 µM [22], and Taq DNA polymerase (Thermo Scientific) 1.25. There were cycles of 95 °C for 2 min, followed by 35 cycles of 95 °C for 30 s, 56 °C for 30 s, extension at 72 °C for 1 min and 72 °C for 10 min.

### 2.5. HBV and HDV Viral Load Quantification

The plasmids pL-HBsAg [27] and pSVL(D3) [28] were (Addgene) quantified by spectrophotometry (NanoDrop 2000—Thermo Scientific), and the number of copies per µL was determined on the NEBCalculator platform (https://nebiocalculator.neb.com/#!/dsdnaamt (accessed on 27 June 2023).

Serial dilutions of 10^8^–10^1^ plasmid copies/µL and 10^5^–10^1^ copies/µL were made for HBV and HDV, respectively. Real-time PCR (qPCR) was performed using SYBR Mix (Thermo Scientific) in a Bio-Rad CFX96 Thermocycler (Hercules, CA, USA), primers S3a and S3as for HBV and primers HDV-E and HDV-A for HDV (Supplementary Table S1). According to the Cq obtained, a linear regression was performed with an R^2^ > 0.95. Following the linear regression parameters, the viral load of positive samples for conventional PCR was quantified.

### 2.6. Phylogenetic Analysis

Positive samples for the viral genome (HBV/HDV) were amplified using the enzyme Taq Platinum (Invitrogen, Waltham, MA, USA). Sequencing of amplicons was performed using the automated Sanger dideoxynucleotide method (BigDyeTM terminator by Macrogen Inc., Seoul, Republic of Korea). The sequences were assembled with the SeqMan, DNASTAR. A data set was designed with the sequences obtained in this study, representative sequences of HBV genotypes and HDV genotypes available in GenBank and sequences from other studies carried out in Colombia. The alignment was performed with Clustal W and Muscle in Mega X.

To determine the best substitution model, MEGA X was used with the higher BIC. Subsequently, phylogenetic analyses were performed using Mr. Bayes. For HBV, we used the model K2+G with MCMC for 1 million generations to have ESS > 200. For HDV, we used the model GTR+G with MCMC for 1.5 million generations to have ESS > 200. The Fig-Tree v1.4.2 program was used to view and edit the phylogenetic trees. For phylogenetic analyses, the HDV sequence MK598012.2 described in rodents was used as an outgroup and 97 sequences obtained from the GenBank of all HDV genotypes were included.

### 2.7. HBV Immune Escape Mutans

Mutations in sequences of HBV PreS1, PreS2 and S regions were identified using Geno2pheno [https://hbv.geno2pheno.org/ (accessed on 27 June 2023)] and Mega X to describe possible immune escape mutants.

### 2.8. Statistical Analysis

Descriptive statistics were carried out using the RStudio v. 2022.02.1+461.

## 3. Results

### 3.1. Socio Demographic Characteristics of the Study Population

Seventy-five hepatitis B cases notified during the period of 2015–2022 in indigenous populations from the study area were included: 49.3% (37/75), 24.0% (18/75), 20.0% (15/75), and 6.7% (5/75) from Amazonas, La Guajira, Guaviare, and Antioquia states, respectively.

The median age was 30 years old (IQR: 27-37 years old) and the proportion of women was 82.7%. The most important ethnic groups in the study population were Tikuna in Amazonas (23/37), Wayuu in La Guajira (18/18), Nukak in Guaviare (11/15) and Embera Katio in Antioquia (2/5) (Table 1).

### 3.2. Serological Markers of HBV Infection

**Figure 1:**
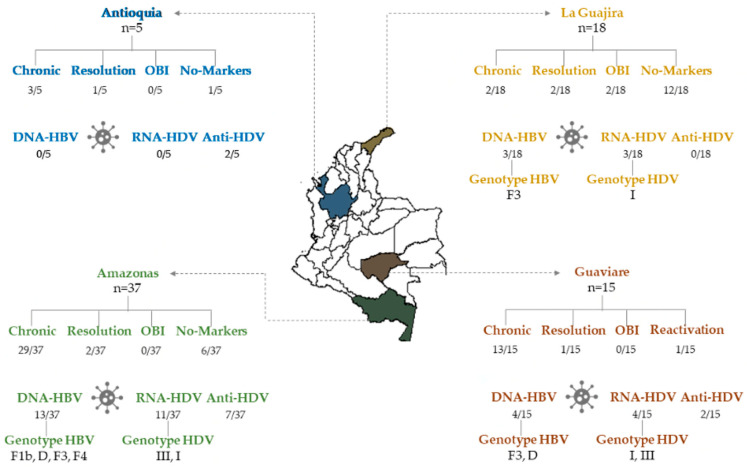
Serological and molecular markers of HBV and HDV infection in indigenous individuals from Amazonas, Antioquia, La Guajira and Guaviare states in Colombia. OBI: Occult HBV Infection.

HBsAg was detected in 64% (48/75) and Anti-HBc IgM or Anti-HBc Total marker in 72% of serum samples of the study population (54/75). According to these results, 48 chronic hepatitis B cases, two cases of OBI and six cases of resolution were characterized. Unexpectedly, 19 notified cases were negative for HBV infection markers (Figure 1 and Supplementary Table S2).

The serological level of AST and ALT were between 34.63–1563 IU/mL and 51.40–494.20 IU/mL in samples obtained from hepatitis B notified cases, respectively. The samples corresponding to the 15 cases with ALT > 49 IU/mL and/or AST > 34 IU/mL serum levels were analyzed for HBeAg and Anti-HBe markers. Of these, one case was positive for HBeAg, ten cases were positive for Anti-HBe and one case was positive for both HBeAg and Anti-HBe. In three cases, the serum sample was insufficient for this analysis.

The profile markers demonstrated one case of reactivation (HBsAg+/Anti-HBc IgM+/Anti-HBc Total+/DNA-HBV+/Anti-HBe+), one case of HBeAg-positive chronic hepatitis B, one case of recurrence of HBeAg and nine cases HBeAg-negative chronic hepatitis B (Figure 1 and Supplementary Table S2).

### 3.3. Detection and Quantification of the HBV Genome

The HBV ORF S region (422–758nt) was amplified in 20/75 (26.67%) samples. In addition, the PreS1 region was amplified in three samples (15%) and the PreS2 region in 6 samples (30%) in 20 of the samples positive for HBV genome detection.

The HBV viral load was >2000 copies/mL in 2 samples and <2000 copies/mL in 14 samples (Supplementary Figure S1). The HBeAg + Chronic Hepatitis B case had a viral load > 2000 copies/mL, and had not received any antiviral treatment. The viral load assay was not successful in four samples probably because the genome copy number was below the limit of detection or due to the viral DNA degradation.

### 3.4. HBV Genotypes and Subgenotypes

**Figure 2:**
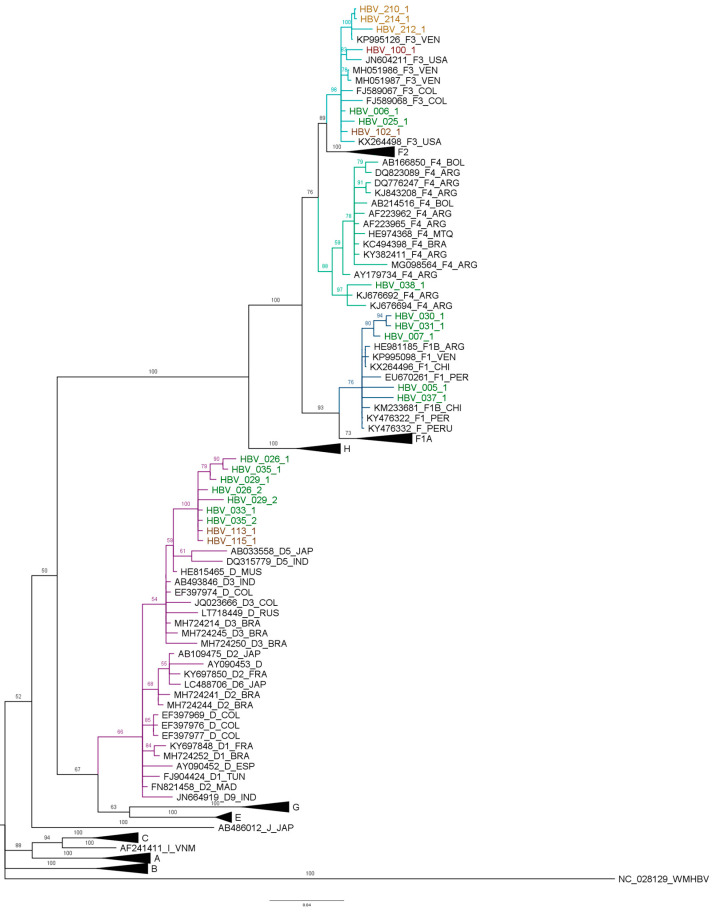
Bayesian analysis of HBV PreS1, PreS2 and S regions. The phylogenetic analysis of the S, S-PreS2 and S-PreS2-PreS1 regions, carried out with Mr. Bayes, K2+G, MCMC 1 million generations and ESS > 200. The study sequences are indicated with a color for each state: Amazonas—green, Guaviare—red and La Guajira—orange. The branches of the F1b subgenotype are marked in dark blue, the F3 subgenotype in light blue, the F4 in green and the D in purple.

The sequences PreS1, PreS2 and S were assembled in Mega X from samples’ successful amplification. Then, the HBV genotype F was characterized in 13 samples by phylogenetic analysis, including five sequences of the subgenotype F1b, seven sequences of the subgenotype F3 and one sequence of the subgenotype F4. Additionally, the description of seven sequences of the HBV genotype D in indigenous populations is significant (Figure 2). However, the subgenotypes for the HBV genotype D were not identified in the phylogenetic analyses because there was not enough resolution.

The five sequences that grouped in the F1b subgenotype belong to Amazonas (005-1, 007-1, 030-1, 031-1 and 037-1). From the seven sequences of the subgenotype F3, three were from La Guajira (210-1, 212-1 and 214-1), two from Guaviare (100-1 and 102-1) and two from Amazonas (006-1 and 025-1). Moreover, there were seven sequences grouped into the genotype D, five were from Amazonas (026-1, 029-1, 033-1, 035-1 and 036-1) and two were from Guaviare (113-1 and 115-1) (Figure 2).

The sequences of the HBV subgenotype F1b from Amazonas were grouped with sequences previously characterized in Colombia, Argentina and Venezuela. Sequence 005-1 is the most distant of this study, and clusters with sequences reported in Argentina. In the subgenotype F3, the 100-1 sequence was grouped in a clade with sequences from Colombia and Venezuela. The sequences from La Guajira (210-1, 212-1 and 214-1) were grouped in the same clade with sequences from Venezuela; the other sequences of the HBV subgenotype F1b from Amazonas and Guaviare (006-1, 025-1 and 102-1) were grouped together with sequences from Colombia and Brazil. 

The Amazonas sequences that grouped with the HBV genotype D are similar to sequences reported in Colombia and in Brazil.

Considering that the HBV subgenotypes F1b and F3 sequences of the study populations do not form a monophyletic clade, it could be suggested multiple introductions. The D genotype sequences of the study form a monophyletic clade different from the other clades of sequences previously described in Colombia; it could also correspond various introductions of HBV D genotype in the country.

### 3.5. HBV Immune Escape Mutants

The HBV sequences were analyzed using the reference genome of HBV NC_003977.2 using the Geno2Pheno program. Although some substitutions were identified, none correspond to HBV immune escape mutants. The Q3L and P36T mutations in the PreS1 region were characterized in one sample from La Guajira. Additionally, A17G, L94Q, Y100C, Y100S, T114P, G145A and W199G mutations in ORF S were detected (Supplementary Table S2). The mutations in positions 100 and 114 are located near the “*a*” determinant [29,30,31].

### 3.6. Serological Markers of HDV Infection

From the 75 cases, 14.67% (11/75) were positive for Total Anti-HDV. One of these corresponded to the reactivation case (HBsAg+/Anti-HBc IgM+/Anti-HBcTotal+/anti-HBe+/DNA-HBV), and the others to chronic HBV infection (HBsAg+/Anti-HBc IgM−/Anti-HBcTotal+) (Supplementary Table S3). The geographical distribution of eleven positive cases for Anti-HDV is shown in Figure 1.

### 3.7. Detection and Quantification of the HDV Genome

From 75 samples, 18 (24%) were positive for the HDV genome. Regarding the serological and molecular profile, 83.33% (15/18) samples had markers of chronic HBV infection (HBsAg+/Total Anti-HBc+); moreover, HBV and HDV genomes were detected in nine samples simultaneously. Additionally, HDV genome was detected in the reactivation case (HBsAg+/Anti-HBc IgM+/Total Anti-HBc+/DNA-HBV+), and in two cases from La Guajira that were negative for markers HBsAg/Anti-HBc. Moreover, one of the cases from La Guajira was positive for the HBV genome, which could correspond to a secondary OBI (Supplementary Table S2). A viral load of >2000 copies of HDV RNA/mL was quantified in the 18 samples (Supplementary Figure S1).

### 3.8. HDV Genotypes

**Figure 3:**
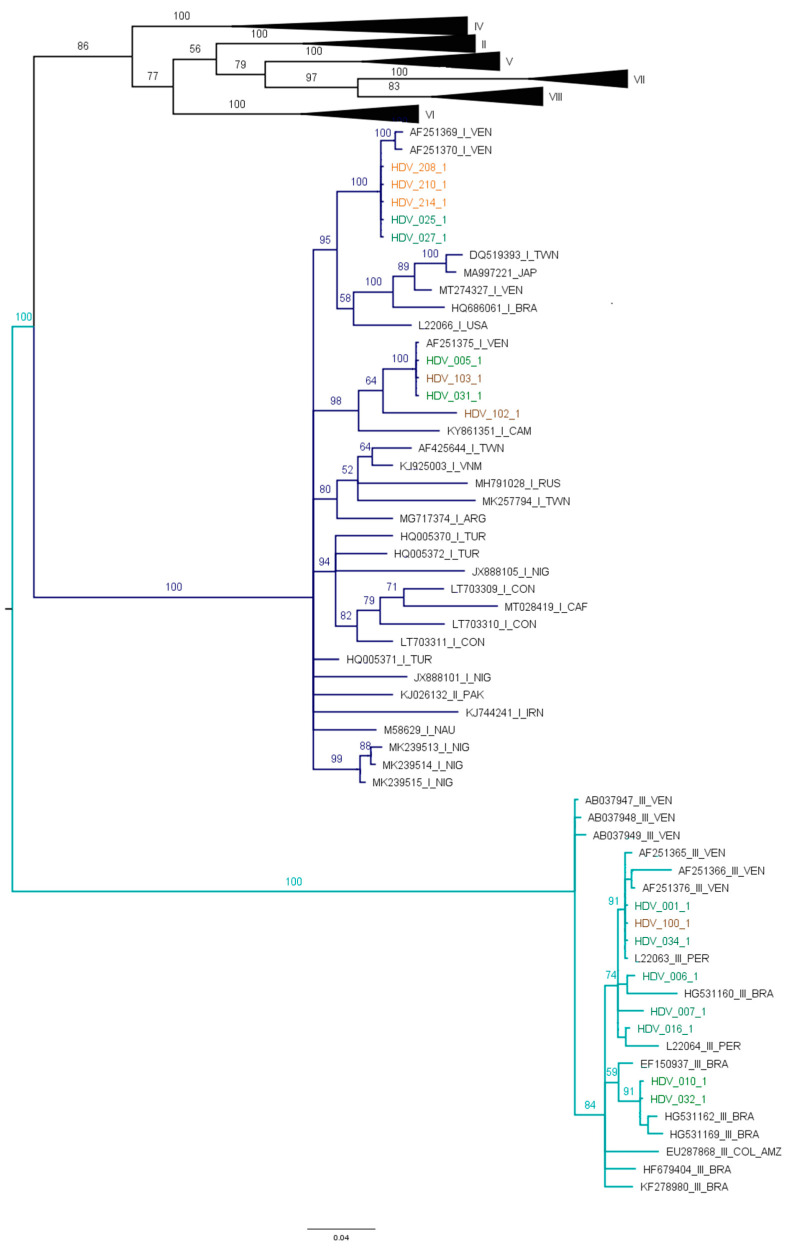
Phylogenetic analysis of HDAg nucleotide sequences. Bayesian analysis was carried out with Mr. Bayes, MCMC 1.5 million generations and ESS > 200. The study sequences are indicated by a color: Amazonas (green), Guaviare (brown) and La Guajira (orange). Branches of genotype I are marked dark blue, with genotype III in light blue. It was determined in the Mega X program that the best substitution model for this dataset is the General Time Reversible (GTR) model with G+I distribution.

Eight sequences of this study were grouped with HDV genotype III, and nine sequences with HDV genotype I (Figure 3).

The sequences of HDV genotype I identified in this study clustered with sequences described mainly in Venezuela and Brazil. Interestingly, in this study, for the first time, the circulation of HDV genotype I in Colombia, including Amazonas (005-1, 025-1 and 027-1), Guaviare (102-1 and 103-1) and La Guajira (208-1, 210-1 and 214-1) states was identified. The sequences that were grouped in HDV genotype III are from Amazonas (001-1, 006-1, 007-1, 010-1, 016-1, 032-1 and 034-1) and Guaviare (100-1), which are similar to sequences reported in Peru, Brazil and Venezuela.

From cases of HBV/HDV infection, HBV F1b/HDV III were identified in 1/9 samples, HBV F1b/HDV I in 2/9 samples, HBV F3/HDV III in 2/9 samples and HBVF3/HDV I in 4/9 samples.

## 4. Discussion

In 1992, the HBV vaccine program was initially implemented in the newborn and child population of Amazonas state in Colombia due to the high prevalence in this region and then introduced to the rest of the country [32]. By 1999, there was evidence of a 70% reduction of HBsAg prevalence in the Amazonas indigenous population. Nevertheless, this region is still endemic for hepatitis B, as described in some studies and in the Colombian epidemiological surveillance system [32,33].

One of these studies was carried out 18 years after the implementation of vaccination in inhabitants of the Amazonas state: 1265 indigenous children (6 months—11 years old) and their mothers [21]. Although the prevalence of HBsAg (0.5%) and anti-HBc (3.6%) in the child population demonstrated the impact of HBV vaccine program, 30.9% of the mothers were positive for anti-HBc and 9% (51/572) were positive for HBsAg; this finding in the group of mothers is characteristic of a high endemicity population. Given the average age of these women (33 years, rank 17–63 years), it was inferred that a proportion of them did not have the opportunity to be vaccinated during their childhood [21]. Recently, an interesting study demonstrated 0% HBsAg prevalence in 3203 children < 10 years of age from 36 high-risk municipalities from across the country, including municipalities of Antioquia, Amazonas, La Guajira and Guaviare. This important result demonstrates the commitment to vaccination coverage in the child population of Colombia and contributes to the WHO Viral hepatitis goal [34].

Therefore, hepatitis B is still a public health problem in different indigenous communities of America, including various ethnic groups in Colombia that are settled not only in the Amazon basin but also in other regions of the country [35].

In this study, 75 cases of hepatitis B, found in indigenous individuals from communities well-established in Guaviare, Antioquia, La Guajira and Amazonas states, were characterized for HBV and HDV infection markers. The cases were notified between 2015 and 2022 to the Colombian public health surveillance system [36].

On average, 2.9% of the total hepatitis B cases notified in the Colombian epidemiological surveillance system during the period 2015–2020 corresponded to the indigenous population [10]. This percentage could be an underestimation of the real situation of hepatitis B in indigenous communities, given the high prevalence of HBV infection described in some studies and the factors that could be related to the vulnerability of these populations, such as geographic isolation, ideology and traditions, as well as the limitations of access to the health system.

Most hepatitis B cases reported in the present study were women (82.7%). Indeed, an important proportion of cases notified to the Colombian epidemiological surveillance system correspond to pregnant women identified through the prenatal control program. Other hepatitis B cases found in the country corresponded to blood donors and viral hepatitis and/or with a diagnosis of end-stage liver diseases.

Hepatitis B cases are notified in the Colombian epidemiological surveillance system using HBsAg marker (Immunoassay or rapid test). In the present study, 48/75 chronic hepatitis B cases were characterized (HBsAg+/Anti-HBc+). Additionally, 8% (6/75) of cases were HBsAg−/Anti-HBc+ classified as OBI (2 cases) and resolution (6 cases). Unexpectedly, 19/75 notified cases were negative for all HBV infection markers, most of them from the La Guajira state (63.16%, 12/19). Despite efforts to elucidate this inconsistency by performing a second analysis of some of these samples, the absence of HBV infection markers was confirmed; moreover, there are limitations of reported cases’ information such as the commercial rapid test used for HBsAg.

The HBV genome was detected in 26.67% (20/75) of samples, including two cases negative for HBsAg that probably correspond to secondary OBI. The low positivity rate of the viral genome detection in this study could be related to viral load below the detection limit or by the down regulation of HBV replication by HDV. Indeed, 18/75 (24%) of cases were positive for HDV infection markers [11].

The HBV genotype F was characterized in 65% (13/20) of samples obtained from these indigenous populations, including five of the subgenotype F1b, seven of the subgenotype F3 and one subgenotype F4. The high frequency of genotype F has been previously reported in different Colombian populations [21,22,37,38]. Moreover, the subgenotype F1b has already been described in two studies in indigenous communities from Amazonas [21,22]. The F3 sequences of the present study were grouped into a clade with other sequences in Colombia characterized mainly in blood donors [8,39,40] and in patients with cirrhosis and/or HCC in the country [9,41]. Peláez-Carvajal et al. also identified the circulation of the subgenotype F3 in Guaviare [37]. Additionally, three cases of the HBV genotype F from La Guajira were grouped with sequences from Venezuela, a country in which the Wayuu indigenous community also lives.

The HBV subgenotype F4 was characterized for the first time in indigenous communities in Colombia. However, the subgenotype has been previously described by the indigenous of the Mbyá-Guaraní community settled in Paraguay, southern Brazil and Bolivia, as well as in other populations in Argentina [38,42].

The HBV genotype D was characterized in two samples from Guaviare and eight samples from Amazonas. Unfortunately,, it was not possible to identify the subgenotypes. This is the first report of the HBV genotype D in indigenous communities in Colombia, although it has been reported previously in blood donors [6,9] and Afro-Colombian populations [43].

Spitz et al. reported the genotype D, and subgenotypes D1, D2, D3 and D4, in different regions of Brazil [44]. Additionally, the spatial-temporal reconstruction of the HBV genotype D carried out by these authors demonstrated that the closest common ancestor for this genotype corresponded to the migrations of Europeans and Arabs in the 19th and 20th centuries in South America [44].

According to the analysis of 20 sequences HBV ORF S obtained from the study population, the mutations Q3L and P36T in PreS1, and A17G, L94Q, Y100C, Y100S, T114P, G145A and W199G in the S domain were identified. However, it is necessary to continue the genomic surveillance, since Jaramillo et al. reported the G145R and W156* immune escape mutants in samples obtained from a mother and a child in Amazonas state, respectively [21]. Furthermore, Peláez-Carvajal et al. reported the P120Q mutation in a sample from Guaviare state, which has been associated with a decrease in HBsAg detection by immunoassay [37].

A high frequency of serological and molecular markers of HDV infection (29.33%) was described in this study. The prevalence of HDV infection by state were 35.13% (13/37) in Amazonas, 26.67% (4/15) in Guaviare, 16.67% (3/18) in La Guajira and 40% (2/5) in Antioquia. In addition, this is the first report of HDV infection in indigenous communities of La Guajira and Antioquia states.

In a previous study carried out in Amazonas state in 2013, the 43.5% (10/23) of samples obtained from indigenous > 18 years old and the HBsAg rapid test + were positive for Anti-HDV antibodies [22]. Moreover, the HDV genome was detected in 7/11 (63.64%) of samples Anti-HDV Total+ [22]. However, in the present study, 61.11% (11/18) of the samples positive for the HDV genome were negative for the Total Anti-HDV. This profile may be due to a limitation of the competitive ELISA kit used (Dia.Pro).

A high prevalence of the HBV/HDV coinfection/superinfection has also been reported in other indigenous communities in Latin America [45]. In Venezuela, the Anti-HDV frequency was among 11.11–60.71% in Yanomami and Piaroa communities that inhabit Orinoquia region, and 50.46% in the Yucpa community [46,47,48]. In Ecuador, an Anti-HDV prevalence of 31.91% was reported in the Waorani community [49]. Meanwhile, in the Amazon basin, an Anti-HDV prevalence of 13.4% and around 2.11–39.02% in indigenous communities from Brazil and Peru, respectively [50,51], has been described.

Among the indigenous with molecular markers of HDV infection, one case (Anti-HBc IgM+/Anti-HBe+/HBV DNA+/Anti-HDV+/HDV RNA+) was identified as a reactivation and superinfection. Further, in 15/22 cases, Anti-HBc IgM−/Anti-HBc Total+/ARN-HDV+ markers were detected corresponding to possible cases of superinfection [52].

Moreover, HBV or HDV genomes were detected in two cases HBsAg−/Anti-HBc− from La Guajira. According to the serological and molecular markers profile, it could correspond to secondary OBI [53]. A similar pattern was described by Chemin et al., in 2/160 samples obtained from patients Anti-HDV+ and markers of HCV infection but without any serological or molecular marker of HBV infection [54].

The HDV genotype III, the most prevalent genotype in South America, was characterized in 8/18 samples in the study population [22,24]. Moreover, the HDV genotype I circulation was described for the first time in Colombia in 10/18 samples. Unprecedented, in 2001, the HDV genotype I was reported in South America in the indigenous population from Perijá region in Venezuela [55]; subsequently, in 2006, this HDV genotype was characterized in samples from indigenous communities settled in Acre and Rondonia regions in Brazil [56,57].

The HDV genotype I introduction in Colombia could be related to the interactions with populations from Venezuela and Brazil. Indeed, the phylogenetic relationships of HBV and HDV with sequences from Venezuela, Brazil, and Peru suggest an interaction of indigenous populations from different ethnicities. This hypothesis has already been suggested by di Filippo et al. [22] and López et al. that have also described relationships between Tikuna communities of Brazil, Colombia and Peru from an ethnic historical and ethnographic analysis [58].

These results, including the first characterization of HBV and HDV genotypes and subgenotypes in these indigenous communities in Colombia, could indicate not only the interaction with nearby populations [58], but also with other Colombian populations and/or individuals from other countries of the region and from other continents through tourist visits.

Despite the important results obtained in the present study, this research had some limitations. There was a bias in representativeness and the number of hepatitis B cases of each state, since it depended on the notification of health institutions to the Colombian epidemiological surveillance system and the accessibility of the study population according to their place of residence, as well as the distance from the capital of each one of the states. Additionally, the clinical information of the study population, necessary for the characterization of the cases, was not available. Finally, the size of the HBV ORF S sequence (336 bp) is a limitation for the phylogenetic resolution to identify subgenotypes with Bootstrap > 70; moreover, the methodologies used for the PreS1 and PreS2 regions had a low efficiency.

## 5. Conclusions

The results obtained in this study provide evidence that HBV and HDV infections are still a public health problem in indigenous communities in Colombia, particularly in those from Amazonas and Guaviare states. In addition, we described a high prevalence of serological or molecular markers of HDV infection (29.33%, 22/75). The circulation of a high diversity of HBV genotypes (F1b, F3, F4 and D), the first report of HDV genotype I circulation in Colombia and also the HDV infection evidence in some of these indigenous communities for the first time are important findings to explore the variables associated to the high prevalence of HBV and the interethnic relationships of these communities. This study demonstrated the importance of addressing this health issue in indigenous communities with an interdisciplinary approach and community-based perspective to strengthen prevention through vaccination, diagnosis and active search for cases, and follow-up.

## Figures and Tables

**Table 1:** Sociodemographic characteristics of the study population.

	Hepatitis B Cases Notified in 2015–2021 n = 75	
**Sex**	No.	%
Female	62	82.7
Male	13	17.3
**Age (Years)**		
Median	30 (IQR 27–37)	
**State/Ethnic groups**		
**Amazonas**	37	49.3
Tikuna	23	30.7
Yaguas	10	13.3
Andoque	2	2.7
Cocama	2	2.7
**La Guajira**	18	24
Wayuu	18	24
**Guaviare**	15	20
Nukak	11	14.7
Jiw	2	2.7
Tukano	1	1.3
Yuruti	1	1.3
Cubea	0	0
**Antioquia**	5	6.7
Embera Katio	2	2.7
Embera *	1	1.3
Embera Chami	1	1.3
Embera Dovida	1	1.3

IQR: interquartile range. *: Not defined.

## Data Availability

The sequences will be available at GenBank: https://www.ncbi.nlm.nih.gov/nuccore (accessed on 27 June 2023).

## Supplementary Materials

The following supporting information can be downloaded at https://www.mdpi.com/article/10.3390/microorganisms11071739/s1, Supplementary Table S1. Primers for HBV and HDV genome amplification Supplementary Table S2. Serological and molecular markers of HBV infection in cases reported in the study population in the period 2015–2022 in Colombia. Supplementary Table S3. Serological and molecular markers of HBV and HDV infection in cases reported in the study population in the period 2015–2022 in Colombia. Supplementary Figure S1. Quantification of HBV and HDV viral load in samples obtained from hepatitis B cases in indigenous population.
