# Supplementary material for: Epidemiology and Genetic Diversity of Hepatitis B Virus and Hepatitis Delta Virus Infection in Indigenous Communities in Colombia
Source: Microorganisms. 2023 Jul 3;11(7):1739. doi: 10.3390/microorganisms11071739 (PMC10386745; doi:10.3390/microorganisms11071739)
Supplement: Supplementary file 1 [file microorganisms-11-01739-s001.zip › microorganisms-2426311-supplementary.pdf]

Supplementary Table S1. Primers for HBV and HDV genome amplification

**Name**	**5´-3´**	**Position**	**Reference**
YS1	GCGGGGTTTTTCTTGT TGA	203-221	Zeng et al., 2004
YS2	GGGACTCAAGATG TTGTACAG	787-767	Zeng et al., 2004
S3s	TGCCTCATCTTCTTRTTGGTTCT	422-444	Schaefer et al., 2003
S3as	CCCCAAWACCAVATCATCCATATA	758-735	Schaefer et al., 2003
P1	TTTTTCACCTCTGCCTAATCA	1823-1843	Gunther et al., 1995
P2	AAAAAGTTGCATGGTGCTGG	1827-1808	Gunther et al., 1995
2440N	TTGAGATCTTCTGCGACGCGGC	2412-2433	Hu et al., 2000
58P	CCTGCTGGTGGCTCCAGTTC	77-58	Hu et al., 2000
P3006f	AATGGCAAACAAGGTAGGAGTGG	194-215	Jaramillo et al., 2017
P213r	AAACACACCGCCTGTAACACGA	2994-2979	Jaramillo et al., 2017
8531U	CGGATGCCCAGGTCGGACC	855-873	Zhang et al., 1996
1302D	GGATTCACCGACAAGGAGAG	1324-1305	Zhang et al., 1996
HDV-E	GAGATGCCATGCCGACCCGAAGAG	885-908	Casey et al., 1993
HDV-A	GAAGGAAGGCCCTCGAGAACAAGA	1290-1267	Casey et al., 1993

The primer positions agree with the HBV and HDV reference genomes NC_003977.2 and NC_001653.2 respectively.

Supplementary Table S2. Serological and molecular markers of HBV infection in the study population (Cases reported in indigenous individuals during 2015-2022 in Colombia)

HBV infection	Sample	State	HBsAg	anti-HBc Total	anti-HBc IgM	ALT (IU/mL)	AST (IU/mL)	HBeAg	Anti-HBe	HBV DNA	HBV Genotype	Immune escape mutants	HDV RNA	HDV Genotype	anti-HDV
Chronic	004-1	AMZ	+	+	-	51.40	63.50	-	+	-	ND	ND	-	ND	-
	011-1	AMZ	+	+	-	7.00	19.10	ND	ND	-	ND	ND	-	ND	-
	014-1	AMZ	+	+	-	27.00	35.10	-	+	-	ND	ND	-	ND	-
	015-1	AMZ	+	+	-	20.10	38.20	-	+	-	ND	ND	-	ND	-
	017-1	AMZ	+	+	-	21.90	21.40	ND	ND	-	ND	ND	-	ND	-
	018-1	AMZ	+	+	-	11.80	20.20	ND	ND	-	ND	ND	-	ND	-
	020-1	AMZ	+	+	-	14.70	21.90	ND	ND	-	ND	ND	-	ND	-
	021-1	AMZ	+	+	-	116.20	88.00	-	+	-	ND	ND	-	ND	-
	022-1	AMZ	+	+	-	17.10	23.20	ND	ND	-	ND	ND	-	ND	-
	026-1	AMZ	+	+	-	14.70	22.60	ND	ND	+	D	-	-	ND	-
	030-1	AMZ	+	+	-	13.20	19.30	ND	ND	+	F1B	-	-	ND	-
	033-1	AMZ	+	+	-	15.40	26.80	ND	ND	+	D	-	-	ND	-
	035-1	AMZ	+	+	-	16.60	22.30	ND	ND	+	D	-	-	ND	-
	036-1	AMZ	+	+	-	83.90	298.90	ND	SND	+	D	-	-	ND	-
	037-1	AMZ	+	+	-	48.40	37.70	ND	ND	+	F1B	Y100S, T114P, G145A	-	ND	-
	038-1	AMZ	+	+	-	14.80	33.30	ND	ND	+	F4	Y100C	-	ND	-
	105-1	GUV	+	+	-	23.30	28.40	ND	ND	-	ND	ND	-	ND	-
	106-1	GUV	+	+	-	5.60	7.50	ND	ND	-	ND	ND	-	ND	-
	107-1	GUV	+	+	-	1.90	9.10	ND	ND	-	ND	ND	-	ND	-
	108-1	GUV	+	+	-	16.70	27.80	ND	ND	-	ND	ND	-	ND	-
	109-1	GUV	+	+	-	9.40	22.70	ND	ND	-	ND	ND	-	ND	-
	110-1	GUV	+	+	-	4.50	10.60	ND	ND	-	ND	ND	-	ND	-
	113-1	GUV	+	+	-	9.40	9.60	ND	ND	+	D	-	-	ND	-
	115-1	GUV	+	+	-	15.10	22.60	ND	ND	+	D	-	-	ND	-
	116-1	GUV	+	+	-	13.30	15.30	ND	ND	-	ND	ND	-	ND	-

	219-1	GUA	+	+	-	11.70	17.96	ND	ND	-	ND	ND	-	ND	-
	302-1	ANT	+	+	-	7.70	13.50	ND	ND	-	ND	ND	-	ND	-
**Resolution**	009-1	AMZ	-	+	-	30.30	27.30	ND	ND	-	ND	ND	-	ND	-
	012-1	AMZ	-	+	-	12.30	19.50	ND	ND	-	ND	ND	-	ND	-
	114-1	GUV	-	+	-	4.30	10.80	ND	ND	-	ND	ND	-	ND	-
	204-1	GUA	-	+	-	21.70	34.90	ND	ND	-	ND	ND	-	ND	-
	304-1	ANT	-	+	-	24.16	18.01	ND	ND	-	ND	ND	-	ND	-
	220-1	GUA	-	+	+	40.40	32.19	ND	ND	-	ND	ND	-	ND	-
**OBI**	212-1	GUA	-	-	-	13.30	23.10	ND	ND	+	F3	-	-	ND	-
**Cases notified without any markers of HBV inf**	002-1	AMZ	-	-	-	6.50	12.50	ND	ND	-	ND	ND	-	ND	-
	003-1	AMZ	-	-	-	9.90	18.40	ND	ND	-	ND	ND	-	ND	-
	013-1	AMZ	-	-	-	40.90	27.90	ND	ND	-	ND	ND	-	ND	-
	019-1	AMZ	-	-	-	19.10	22.60	ND	ND	-	ND	ND	-	ND	-
	024-1	AMZ	-	-	-	32.40	38.30	ND	ND	-	ND	ND	-	ND	ND
	027-1	AMZ	-	-	-	10.30	21.80	ND	ND	-	ND	ND	-	ND	-
	200-1	GUA	-	-	-	21.60	26.10	ND	ND	-	ND	ND	-	ND	-
	201-1	GUA	-	-	-	39.60	59.80	ND	ND	-	ND	ND	-	ND	-
	202-1	GUA	-	-	-	11.30	19.30	ND	ND	-	ND	ND	-	ND	-
	203-1	GUA	-	-	-	20.00	17.00	ND	ND	-	ND	ND	-	ND	-
	213-1	GUA	-	-	-	35.50	39.00	ND	ND	-	ND	ND	-	ND	-
	215-1	GUA	-	-	-	8.20	13.91	ND	ND	-	ND	ND	-	ND	-
	216-1	GUA	-	-	-	38.80	34.63	ND	ND	-	ND	ND	-	ND	-
	217-1	GUA	-	-	-	17.00	26.31	ND	ND	-	ND	ND	-	ND	-
	218-1	GUA	-	-	-	24.10	32.52	ND	ND	-	ND	ND	-	ND	-
	221-1	GUA	-	-	-	48.60	57.88	ND	ND	-	ND	ND	-	ND	-
	222-1	GUA	-	-	-	6.20	17.87	ND	ND	-	ND	ND	-	ND	-
	301-1	ANT	-	-	-	18.50	23.50	ND	ND	-	ND	ND	-	ND	-

AMZ: Amazonas. ANT: Antioquia. GUA: Guajira. GUV: Guaviare. ND: Not Defined. OBI: Occult HBV Infection.

Supplementary Table S3. Serological and molecular markers of HBV/HDV co/super-infection in the study population (Cases reported in indigenous individuals during 2015-2022 in Colombia)

HBV/HDV co/super-infection	Sample	State	HBsAg	anti-HBc Total	anti-HBc IgM	ALT (IU/mL)	AST (IU/mL)	HBeAg	Anti-HBe	HBV DNA	HBV Genotype	Immune escape mutation	HDV RNA	HDV Genotype	anti-HDV
Chronic	001-1	AMZ	+	+	-	27.30	42.20	+	+	-	ND	ND	+	3	+
	005-1	AMZ	+	+	-	7.40	15.00	ND	ND	+	F1B	L94Q	+	1	-
	006-1	AMZ	+	+	-	8.90	19.00	ND	ND	+	F3	-	+	3	-
	007-1	AMZ	+	+	-	6.00	15.20	ND	ND	+	F1B	-	+	3	-
	010-1	AMZ	+	+	-	13.40	41.50	-	+	-	ND	ND	+	3	+
	016-1	AMZ	+	+	-	54.80	140.40	-	+	-	ND	ND	+	3	+
	023-1	AMZ	+	+	-	38.20	32.10	ND	ND	-	ND	ND	-	ND	+
	025-1	AMZ	+	+	-	36.90	50.90	ND	ND	+	F3	W199G	+	1	-
	028-1	AMZ	+	+	-	9.00	22.20	ND	ND	-	ND	ND	+	1	-
	029-1	AMZ	+	+	-	29.50	48.90	ND	ND	+	D	-	-	ND	+
	031-1	AMZ	+	+	-	16.10	21.90	ND	ND	+	F1B	-	+	1	-
	032-1	AMZ	+	+	-	42.20	45.20	-	+	-	ND	ND	+	3	+
	034-1	AMZ	+	+	-	494.20	1562.70	-	+	-	ND	ND	+	3	+
	102-1	GUV	+	+	-	57.10	35.50	-	+	+	F3	A17G	+	1	-
	103-1	GUV	+	+	-	18.80	21.50	ND	ND	-	ND	ND	+	1	+
	111-1	GUV	+	+	-	19.00	23.80	ND	ND	-	ND	ND	+	ND	-
	210-1	GUA	+	+	-	36.90	37.00	+	-	+	F3	Q3L (PreS1), P36T (PreS1)	+	1	-
	300-1	ANT	+	+	-	7.70	14.80	ND	ND	-	ND	ND	-	ND	+
	303-1	ANT	+	+	-	21.97	8.55	ND	ND	-	ND	ND	-	ND	+
Reactivation	100-1	GUV	+	+	+	53.40	131.70	-	+	+	F3	-	+	3	+
OBI	208-1	GUA	-	-	-	13.70	17.10	ND	ND	-	ND	ND	+	1	-
	214-1	GUA	-	-	-	23.90	28.10	ND	ND	+	F3	-	+	1	-

AMZ: Amazonas. ANT: Antioquia. GUA: Guajira. GUV: Guaviare. ND: Not Defined. OBI: Occult HBV Infection.

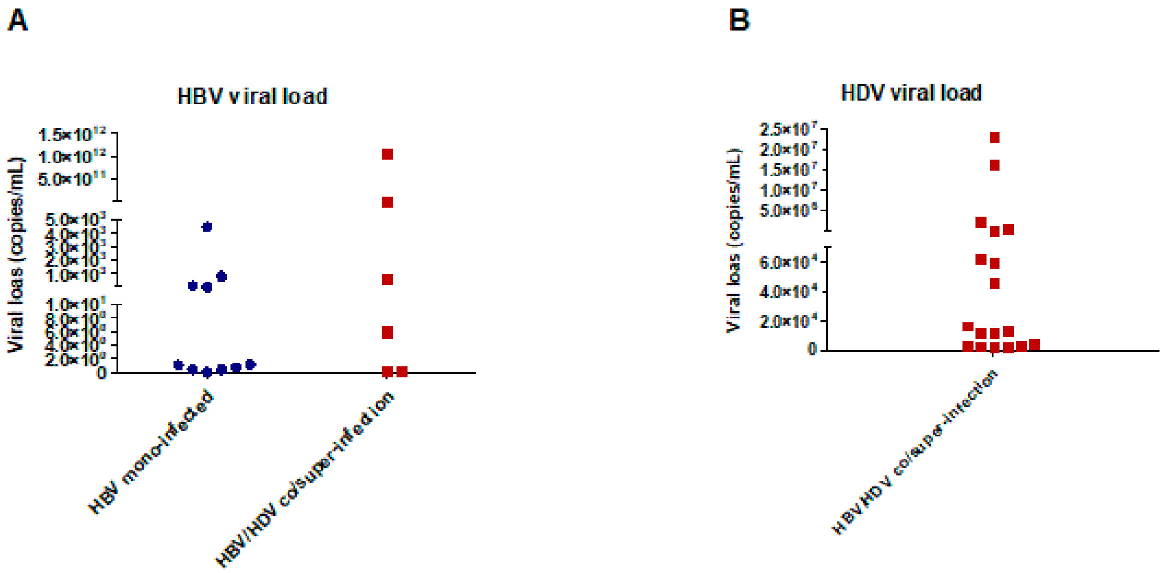

Supplementary Figure S1. Quantification of HBV and HDV viral load in samples obtained from Hepatitis B cases in indigenous population. **A**. HBV viral load in positive serum samples for the detection of the HBV genome, ORF S. **B**. Quantification of HDV viral load in positive serum samples for HDV genome detection. Each sample is analyzed by qPCR in triplicate and the average of the two most homogeneous Cq is taken into account.
